# Our Journal for 1879

**Published:** 1878-12-20

**Authors:** R. C. Word

**Affiliations:** Managing Editor


### OUR JOURNAL FOR 1879.

An improvement in form and some addition to reading matter will be made in our next volume, commencing with our January issue. Our motto is onward and upward. We have perfected arrangements for printing which will insure a more successful and punctual publication of the journal than heretofore. These improvements will add considerably to our expenses, and we shall therefore expect our readers to show their appreciation of our efforts by promptly settling up dues unpaid, and by renewing their subscriptions.

We take for granted that every one of our present subscribers will renew his subscription, and unless notified not to do so before the mailing of our next number (January 20th), we shall enter their names upon the list for 1879. Let our friends stick to us, and THE RECORD, so useful and practical in the past, will be made more so in the future.

R. C. WORD,
Managing Editor.

